# Investments

**Published:** 1912-07-15

**Authors:** Charles G. Daeves

**Affiliations:** Chicago


### INVESTMENTS.

BY CHARLES G. DAEVES OF CHICAGO.

Extracts from an address before the Chicago Dental Society and printed in the DENTAL REVIEW for July.

### DEALING WITH THE STOCK EXCHANGE.

The ordinary man has not one chance in a hundred of making money in stock speculation. Now, why hasn't he?

In the first place, the investor in stock on the exchanges, in ninety cases out of one hundred, invests upon representations which he does not or cannot investigate. He does not know the conditions which surround the investment which he is asked to make. He is buying goods from a public show window, managed by brokers and agents with the seller hiding in the dark.

We all naturally want what seems hard to get and we become interested in anything which promises to give us a profit which is generally something which is increasing in price before our eyes, the presumption being naturally that it will continue to increase in price. We will say that some of these manipulators of the stock exchange want to get some of the money of the dentists or the physicians whose attention is chiefly devoted to their work and whose professional proficiency comes at the expense of the time which is requisite to enable them to invest their money in the best possible way. So a number of interested brokers get together and manipulate some stock which has a market value up or down until the public get interested in it and then they unload it at a fancy price on the people who will not take the trouble to investigate its real merits.

### THE BOARD OF TRADE.

I do not know whether any of you are smart enough to beat this game. I never felt that I was, and I have never tried it. And the lesson to be gained from this is that, when you go on the exchanges, in nine cases out of ten you are playing another man's game. It does not mean that you can't sometimes buy cheap on the stock exchange. At times you can. At times, with a largely scattered stock, an immense crowd of outsiders get frightened and throw it on the exchange, and then values sink away below what they should be. After a pool has sold out at high prices, it is a favorite pastime to create a panic and buy stock back at low prices to form a pool to sell it over again at high prices.

I have seen many people who pride themselves upon their judgment in the matter of stock quotations, of the fluctuations but I have yet to see the broker—and I have done business with a great many of them in loaning money upon their collaterals and stock exchange securities which they bring in —I have yet to see the broker who would say that over five percent. of his customers ever did a continuing business with him and came out with a profit. The financial mortality of men who speculate in the stock exchange—outsiders—is almost as great as the mortality financially among the people who invest in stocks through newspaper advertising. Don't speculate on the exchanges.

### ADVERTISED INVESTMENTS.

I have no doubt that there are many men to whom I am talking in this room who have bought and lost upon stocks through newspaper advertisements. Now, if anybody has ever got out of such stocks more than interest—there are some preferred stocks which have survived and paid ordinary dividends—if anybody ever made an extraordinary profit out of a stock bought through newspaper advertisements, I have yet to see him, and I have seen many investors. So do not buy investments which are advertised in newspapers offering extraordinary returns.

Now this does not at all apply those advertisements of investments in stable securities which give the facts and the statistics bearing upon their safety, the names of the men associated with the enterprise which throws light upon the question of responsible management, and only promise a safe, reasonable and proper return upon money. Municipal bonds, the high-grade public utility bonds offered by responsible banks are of course safe. I am referring to those investments advertised to pay extraordinary returns. Such as you see advertised in conspicuous type in the Sunday newspapers.

### REAL ESTATE INVESTMENTS.

Beware of stock in high office buildings. I suspect that the high office buildings of New York have been one of the greatest sink-holes for the loss of money that they have had down there, for when a change in the form of a safe investment means a permanent lowering of the rate of return, other things being equal, it means a loss of wealth. Now, how opposed to the ordinary idea that statement is to one who has not studied the question. The most of us look at these great office buildings, with their great rental returns, as most profitable investments. We constantly hear about the great success the Rookery has been; of the success of the Railway Exchange Building, and some other buildings, their large rate of investment return generally depending upon some special condition or some especially favorable purchase, or lease of the ground. I am not speaking of exceptionally low leaseholds, and I am not speaking of those forms of city investment which the shrewd man and which the adroit man and the courageous man every time will make better than you and me, but I am speaking of the ordinary building erected upon leased ground, the lease taken at the ordinary current rental rate. I would not buy any stock in any such office buildings.

### REAL ESTATE MORTGAGE LOANS.

The real estate mortgage loan is a good thing. Of course one has to be careful. The rule that the insurance companies sometimes put in force as to farm loans is a very good one, which is to loan forty percent. of the value of the land exclusive of buildings. Well selected first mortage real estate loans are a very good form of investment and a man can very safely realize five per cent. on them—very seldom over five per cent. But be careful of suburban real estate loans and second mortgages, because suburban values are not so stable and most of the money that is lost on city real estate loans is lost on suburban mortgages and second mortgages.

### SAFE INVESTMENTS.

If you are going to try to make over five per cent., invest your money with the man who is a success in the business in which he has succeeded. Now that is not an easy thing to do always. In the first place, the large business man who has succeeded, when he wants additional capital in his business borrows it and gets it at a low interest rate. It is too expensive for him to take small amounts of money as capital in his business and divide up a portion of his profit It is very hard to make that sort of an investment. It can be made at times, and it is about the only way, gentlemen—it is about the only way that I know that the ordinary outside investor can hope to make more than a reasonable five per cent. return, and then he has got to take his chances, because if there is one fixed and unalterable rule in business, it is that the usual rate of compensation for capital varies with the risk.

PUBLIC UTILITIES.

I think well of public utilities. Of course, in that business again there has been some tendency to get away from real values through excessive stock issues and the impression of values which can be created by the payment of too large dividends. The personal equation comes in somewhat as to whether the men in charge of the business are honest. Of course, there is a chance to get fooled on the public utility business, but when it comes to losses it is well to remember that as a result of the panic of 1893 over fifty per cent. of the railroad capitalization of the country went under receivership, while less than one per cent. of public utility capitalization did so. The difference may not be so marked next time, because there has been some improper exploitation.

WHAT IS A SAFE BANK

How is the public to distinguish between honest and dishonest banks? You can tell sometimes, as one of my bank examiners said, when aked the same question. He said, "When I come near a cheese box I can tell whether it contains limburger or not." (Laughter.) In the city of Chicago a man is pretty safe in his banking relations. After the Walsh failure the clearing house banks got together and they said, "Now, we won't trust to the public to watch us alone we will watch each other." Every bank in the city of Chicago every year has one or two examinations by the authorities, it has at least one by the clearing house authorities besides whatever examination it makes by order of its Board of Directors.

And then another thing about the banks of Chicago. The banks of Chicago, as a rule, are loaning against commodities in process of consumption. We have not had here until within the last few years, the vast accumulation of surplus—we are beginning to get it now—that they have had for very many years in the Eastern cities, and in the Eastern cities they loan, somewhat more than we do, against fixed values as distinguished from quick assets. They have built our railroads and mills and things of that sort. We are loaning very largely against grain and packing house products and to manufacturers on cash assets and merchandise in process of consumption. The only way that you can tell an honest man that I know is by his past record and by the way he treats you. It is pretty hard for a man to be dishonest in the banking business in Chicago. I think most of them have moved out.—*Review.*

